# Case series: Five pediatric germ cell/sex cord stroma tumors

**DOI:** 10.1016/j.amsu.2018.11.011

**Published:** 2018-11-28

**Authors:** James G. Glasser, James M. Nottingham, Michael E. Haney, Elizabeth A. Manci

**Affiliations:** 1 Children's and Women's Hospital, University of South Alabama, School of Medicine, Mobile, AL, 36604, United States; 2 University of South Carolina School of Medicine, 2 Richland Medical Park, Suite 300, Columbia, SC, 29203, United States; c USA Children's and Women's Hospital, Mobile, AL, United States

## Abstract

• This report consists of five pediatric tumors of ovarian cell lineage.
• These unusual, interesting tumors challenge both surgeon and oncologist.
• Some appear malignant, seemingly require chemotherapy, but behave with benignity. Some are easily resected; others demand the utmost skill.
• Complexity perplexes; elucidation is by selection – ignoring some things, attending to others.
• Case studies illustrate – for good or ill – the process whereby clinical conundrums are resolved.

Usually, a “case series” consists of a group of patients with nearly identical problems. The purpose of the report is to demonstrate a particularly advantageous diagnostic test or therapy. This report consists of five children with disparate ovarian tumors, each of which is unusual and interesting. (see Tables 1, 2).

**Table 1:** Case series.

Case	Clinical Setting	Tumor Histology	Anatomic Location	Diagnostic Challenge	Therapeutic Difficulty	Interesting Features
1	Puberty	Grade III Stage III **Immature** **Teratoma**	Right Ovary with *Gliosis Peritonei* Tumor Implants Carpeted the Pelvis	Multiply Recurrent Despite Evidence of Maturation Why?	Neovascularity, Utility of Chemotherapy in Treating Pediatric **IT**	Unrecognized Metachronous, Contralateral **MT**
2	Puberty	Stage II **JGCT**	Right Ovary -Capsular Tear – Tumor Spillage	Ollier's Syndrome Versus Bone Metastasis	Omental Neovascularity, An Indication for Chemotherapy?	Tumor's Behavior Belied its Gross Appearance
3	Infancy	Stage II **MT/YST**	Fundus of Stomach	Anemia from Gastric Hemorrhage/Perforation	Tumor Recurrence: **YST**, **IT**, or **MT**? Chemotherapy for Islands of **YST?**	Abdominal Teratoma's Unpredictable Anatomy
4	Infancy	Grade I Stage I, **IT**	Small Bowel Mesentery	Mesenteric Cyst Versus Teratoma	Grade I **IT**	Difficulty in Anticipating Surgical Pitfalls
5	Infancy	Stage IV **Chorio-carcinoma**	Skin/Lungs	Mistaken as Abscess, then as Vascular Malformation	Diagnostic Ambiguity Leads to Therapeutic Inaccuracy	A Biopsy to Ascertain Tissue Diagnosis Is Crucial.

Glossary of Terms.

**MT** – mature teratoma, **IT** – immature teratoma, **JGCT** – juvenile granulosa cell tumor, **YST** – yolk sac tumor.

**Table 2:** Definition of terms [1,2].

Stage 1: Unilateral Tumor Completely Resected	Grade 1: <1 Foci of Immature Teratoma/Low Power Field (Microscopic)
Stage 2: Incomplete Resection with Microscopic Residual	Grade 2: 1–3 Foci of Immature Teratoma/Low Power Field (Microscopic)
Stage 3: Gross Residual Tumor with Spread to Contiguous Organs (LN's, Omentum, Ascites)	Grade 3: >3 Foci of Immature Teratoma/Low Power Field (Microscopic)
Stage 4: Distant Tumor Spread/Liver, etc.	

What makes for an interesting case?

∗ It occurs infrequently.
∗ It is intellectually or technically challenging.
∗ It is puzzling; the denouement is not immediately apparent; it is rather surprising, even counter-intuitive.

Interesting cases are educational; they pique our curiosity; and they are memorable!

The first two cases in this series are adolescent girls with huge ovarian tumors. The resections were immensely challenging, which is unusual in itself! Salpingo-oophorectomy usually proceeds uneventfully; these tumors had parasitized the omentum, and their mobilization required division of fragile blood vessels so numerous as to resemble hydras! Both tumors appeared malignant, but the clinical outcome belied the surgeon's prognostication.

The third case is an infant who presented with a hugely distended abdomen and GI bleeding (his hemoglobin and hematocrit were 2.7 gm% and 12.2%); the etiology was a teratoma that had eroded into his stomach.

The fourth case is a newborn with an abdominal mass, diagnosed (by MR) as a mesenteric cyst. Actually, it was an Immature Teratoma arising from the small bowel mesentery.

The last case is a newborn who presented with a raised, erythematous swelling in her right cheek. Was it an abscess, a vascular malformation, or a tumor?

## 1 Ovarian tumors are complex and confusing!

They are rare; the incidence of ovarian masses is only 5/100,000 girls/year; half are neoplastic; half are cystic. Only 1% of childhood cancers are ovarian; and only 1% of ovarian malignancies occur during childhood. Ovarian tumors are less frequent in young children, but the incidence of malignancy is greater. Epithelial tumors are more common in older women, and the prognosis in adults is worse, because they present with more advanced disease, and adenocarcinoma is less responsive to chemotherapy (Tables 3, 4) [1].

**Table 3:** Overview of ovarian tumors in children.

Frequency	Tumor Derivation	Classification	Marker	Radiographic Appearance
75%	**Germ Cell** Undifferentiated: Differentiated: 1. Embryonic 2. Extra-embryonic	Dysgerminoma Mature Teratoma Immature Teratoma Embryonal Cell Yolk Sac Choriocarcinoma	LDH AFP bHCG AFP bHCG	Solid with Fibro-vascular Septae MT: Cystic, Fat, Ca++ IT: Heterogeneous Solid Heterogeneous Vascular
45%	**Pure:** One Germ Cell Type	25% YST 18% Dysgerminoma 2% Choriocarcinoma		
55%	**Mixed** Multiple Germ Cell Types	30% Teratoma + YST 10% Teratoma + Others^a^ 13% Multiple Non-Teratoma^a^ 2% Gonadoblastoma + Others^a^		

10%	**Sex Cord Stroma**	Granulosa-Theca Sertoli-Leydig	Inhibin	Multi-cystic, Irregular Septa	
15%	**Epithelial**	Serous or Mucinous, Adenocarcinoma	CA 125		

a Dysgerminoma, Embryonal Cell or Choriocarcinoma.

**Table 4:** When is an ovarian mass malignant or benign [3]?.

Likely Malignant	Cystic versus Solid?	Size: < or >9 cm	Markers: AFP/BHCG
High	Solid	>9 cm	+
Intermediate	Cystic or Heterogeneous	>9 cm	–
Low	Cystic	<9 cm	–

AFP < 10 ng/mL is normal.

AFP >1000 ng/mL indicates malignancy and demands more aggressive management [2].

Characteristics of Teratomas (Tables 5, 6) [[4], [5], [6], [7], [8]]:

• They are heterogeneous and contain areas of fat density and calcification.
• They are lobulated, smooth, and encapsulated.
• All 3 embryonic layers (endoderm, mesoderm, and ectoderm) are represented.
• The incidence of teratomas peaks in late adolescence. Most are mature; immature teratomas comprise only 1% of ovarian tumors.
• 20% Teratomas are bilateral, either metachronous or synchronous.
• 10% are immature (neuroepithelial cells) or have a malignant component, YST.
• The terms *mature/immature* are analogous, but without the same connotation, as *differentiated/undifferentiated, benign/malignant.*
• *Gliosis peritonei*, associated with mature teratomas, denotes intraperitoneal spread of tumor, yet it is benign.

**Table 5:** Age adjusted incidence of teratomas.

Age in Years	Occurrence
0–5	10%
5–10	20%
10–15	70%

**Table 6:** Anatomic distribution of childhood teratomas.

Frequency	Site
45%	Sacrococcygeal
27%	Ovary
5%	Testes
6%	Mediastinal
4%	Retroperitoneal
5%	CNS
6%	Cervico-facial
1%	Pericardial
1%	Gastric, Hepatic, Umbilical

Embryogenesis of Teratomas:

• Post meiotic germ cells migrate from the yolk sac and allantois along the dorsal mesentery to the genital ridge, guided by cKit receptors and stem cell factors.
• Half of germ cell tumors (GCT) are ovarian; the other half is dispersed widely, because of aberrant or arrested migration.

The incidence of Juvenile Granulosa Cell Tumors peaks during childhood. These tumors grow rapidly, becoming quite large, but behave with moderate to low grade virulence. Complete resection yields an overall survival of 95% in children who are less than 10 years old, even if the tumor ruptures.

JGCT derive from uncommitted mesenchymal stem cells located beneath the urogenital ridge. Sex cord stroma cells are hormonally active, secreting estrogen or testosterone and suppressing the release of gonadotropins. Children present with increased girth, precocious puberty, menstrual irregularities, galactorrhea, or virilization. Inhibin B and antimullerian hormone values are proportional to follicular growth. Paraneoplastic release of parathyroid hormone may cause elevation of serum calcium. Cytogenetic aberrations, such as chromosome deletions and tumor suppressor gene mutations, have been identified in these tumors. Cisplatin, Etoposide, and Bleomycin are utilized in patients with advanced disease: tumor that is unresectable or recurrent, with high mitotic activity or nuclear atypia, or with ascites [[9], [10], [11], [12]].

Syndromic Sex Cord Stroma Tumors usually occur during the first decade of life, and these tumors are benign, as in Case #2.

Ollier's Syndrome (Enchondromatosis) is a non-hereditary syndrome of mesodermal dysplasia. Fragments of the epiphyseal plate are incorporated into growing bone, forming enchondromas. The deranged cartilage may undergo a malignant transformation to chondrosarcoma (Table 7).

**Table 7:** Syndromes associated with sex cord stroma tumors.

Syndrome	Clinical Characteristics	Associated Tumors
Peutz Jegher	Perioral Melanin Pigmentation, Intestinal Polyps	JGCTG, AnnularTubules,Cystadenoma
Maffucci	Subcutaneous Hemangiomas	JGCT, Fibrosarcoma
Ollier's	Enchondromas, Unilateral Leg Length Disparity	JGCT, Sertoli Leydig Cell Tumors

## 2 Materials and methods

This report was prepared in accord with the PROCESS criteria [13]; it consists of five case histories from the principal author's pediatric surgical practice; three children were treated at USA Children's and Women's Hospital, Mobile, AL; and two were treated at Palmetto Health Children's Hospital, Columbia, SC.

Case 1 Stage 3, Grade 3 Immature Teratoma

**Fig. 1:**
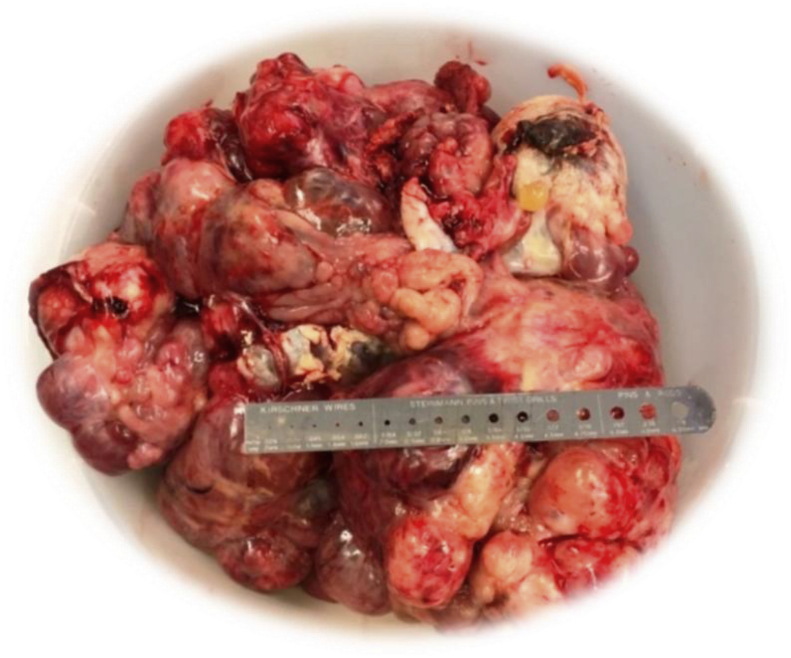
Operative specimen.

**Fig. 2:**
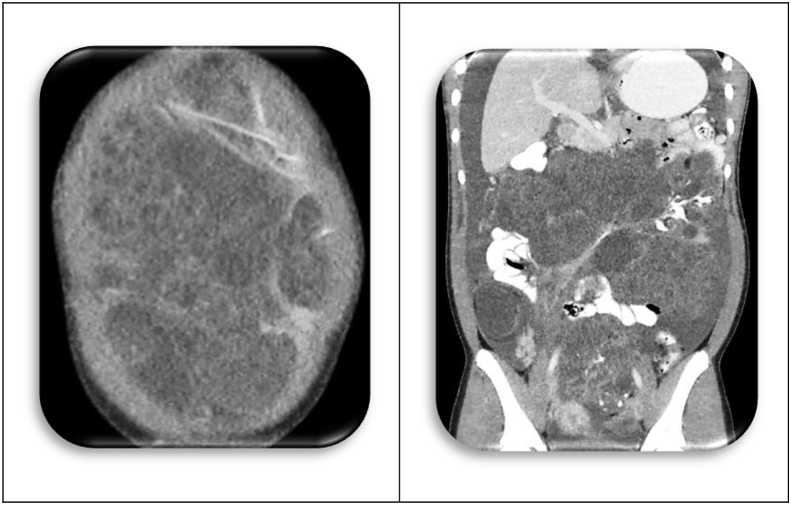
CT scan of Original Tumor.

Case 1 is a 12 years old, pubertal young lady who presented with abdominal distension, so massive that her parents thought she was pregnant (Fig. 1)! A CT scan disclosed an ovarian tumor that had areas of calcification and fat, which is consistent with a teratoma. The demarcation between the MT and IT is apparent in the operative specimen and on the CT scan. It is as if the immature component burst forth with explosive tumor growth from the confines of the well encapsulated (Fig. 2). MT. Surgery revealed widespread *gliomatosis peritonei* with carpeting of the pelvic peritoneum, including the serosal surface of the sigmoid colon. Pathology reported 20% Immature Teratoma (Figs. 4, 5)

**Fig. 3:**
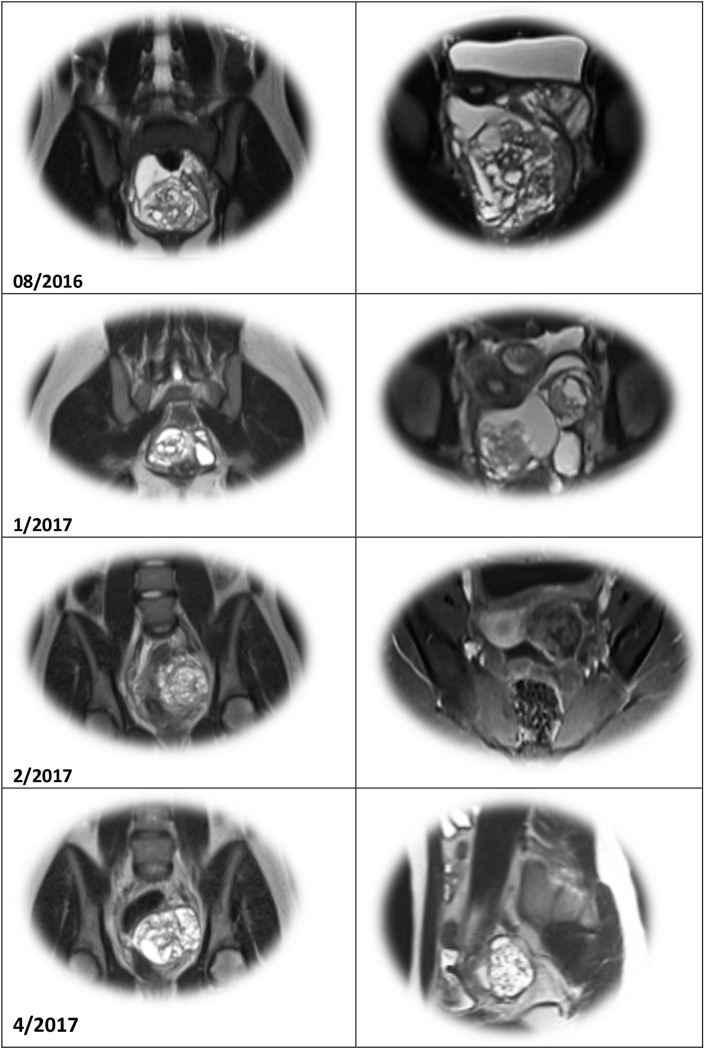
Serial MR's of “recurrent” tumor.

**Fig. 4:**
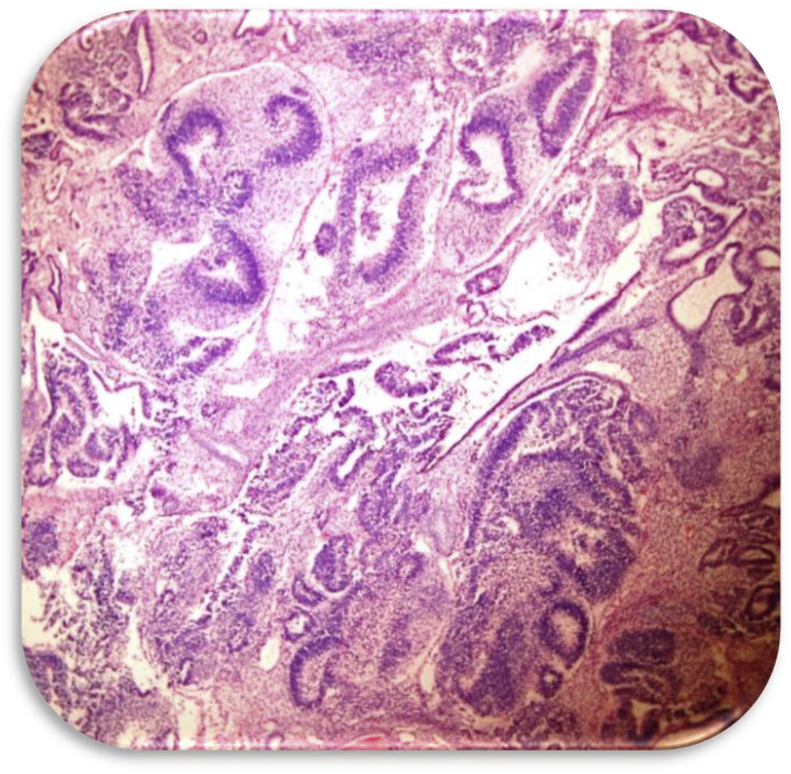
High grade immature teratoma with multifocal primitive neuroepithelium (H&E Zeiss 200x).

**Fig. 5:**
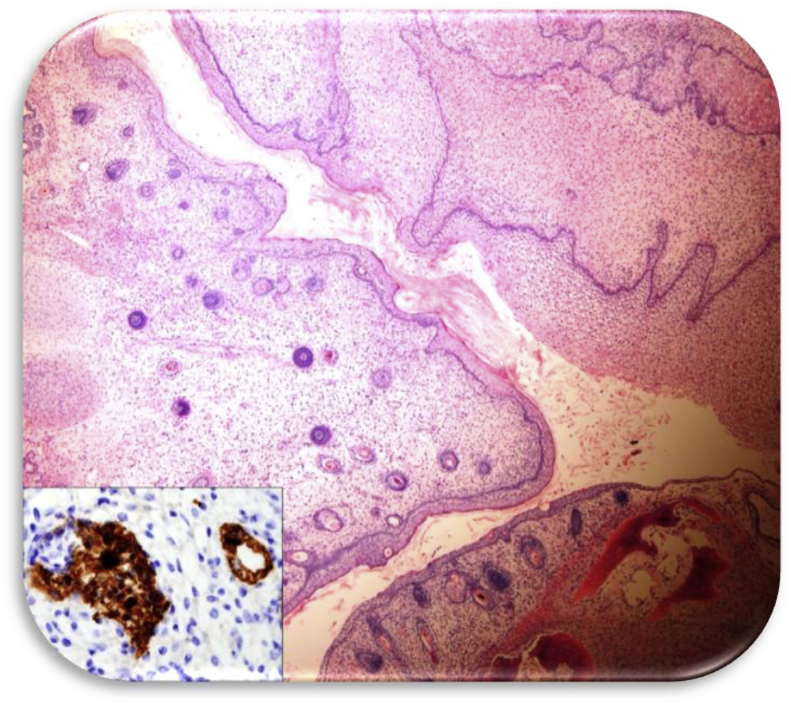
Mature teratomatous elements (including skin, hair follicles, bone, and cartilage) with focal YST (H&E Zeiss 200x) inset: Staining positive for alpha fetoprotein (AFP Zeiss 400x).

### 2.1 Treatment milestones

Even though there was gross residual tumor, the oncologists advised a “wait and see” approach [14]. And predictably, the tumor recurred. Since Teratomas may be mixed (Table 3), careful evaluation of the re-operative specimen for possible malignant elements was advised. None were identified; however, the proportion of Immature Teratoma had diminished to 15%.

An MR was obtained four months later; and again, tumor was present. Surprisingly though, the operative procedure was technically easier. The tumor appeared contained, less aggressive; and pathology review corroborated this impression (Fig. 3). Only 5% of the tissue was IT. The left ovary was distorted and cystic but uninvolved by tumor. The clinicians were optimistic, then baffled by the imaging studies that appeared to show “recurrent” tumor. The oncologists began chemotherapy (Bleomycin, Etoposide, and Cisplatin) hoping to hasten maturation of the teratoma. Repeat imaging was also disappointing; the tumor appeared to be enlarging, rather than diminishing in size. Why? Had chemotherapy created a “growing teratoma?” The only hope seemed to be radical extirpation of this stubborn tumor, but the operative specimen revealed a metachronous, contralateral Mature Teratoma (Tables 8, 9) [8]!

**Table 8:** Summary of imaging studies.

Date	Size of Mass	Description	MR Interpretation
01/04/2017	9.4 × 8.1 × 6.5 cm	Complex Multi-cystic Mass in Rectovaginal Space	Recurrent IT
01/30/2017	5.3 × 5.2 × 5.1 cm	Complex Multi-cystic Mass of Left Adnexa	Recurrent IT
04/14/2017	6.9 × 5.9 × 5.4 cm	Growth of Cystic, Fatty Components of Left Adnexa	Recurrent IT

**Table 9:** Chronology of tumor histology.

Date	AFP	Size of Tumor	Pathology
04/2016	82.9	24 × 30 × 18	Mature Teratoma Right Ovary Containing Brain, Choroid Plexus, Kidney, Respiratory Epithelium, Adipose, Skin with Hair, Hyaline Cartilage 16% Immature Teratoma (High Grade Neuroepithelium) in tumor 100% Immature Teratoma in Gliomatosis Peritonei 20% Immature Teratoma in Pelvic Implants + Ascitic Fluid with Abundant Atypical Cells
08/2016	57.5	9 × 9 × 9.6	Recurrent Teratoma (15% High Grade Neuroepithelium)
01/2017	21	9.4 × 8 × 6.5	Recurrent Teratoma (5% High Grade Neuroepithelium)
03/2017	8.2		
06/2017	3.3	7 × 6 × 5.4	Ovarian Follicles (Contralateral) and Mature Teratoma + Gliomatosis Peritonei, 0% IT

## 3 Discussion of case 1

Principles Guiding the Treatment of Immature Teratoma [2,14]:

1. Grade of tumor is the most significant prognostic variable; tumor Stage is next (see Addendum)
2. Adults with high grade Immature Teratoma receive chemotherapy but may still relapse.
3. Children with completely excised Immature Teratomas are not treated with chemotherapy, even in tumors containing foci of Yolk Sac Tumor.
4. Chemotherapy has no proven benefit in treating children with Grade III, Stage III Immature Teratoma; there evidence that it hastens the maturation of IT.
5. Chemotherapy is utilized only in desperate cases, where extirpation is not feasible.
6. The “growing teratoma syndrome” occurs when chemotherapy destroys the malignant cells (YST) while growth of immature neuroepithelial cells continues unabated.

Case 2 Juvenile Germ Cell Tumor Associated with Ollier's Disease

**Fig. 6:**
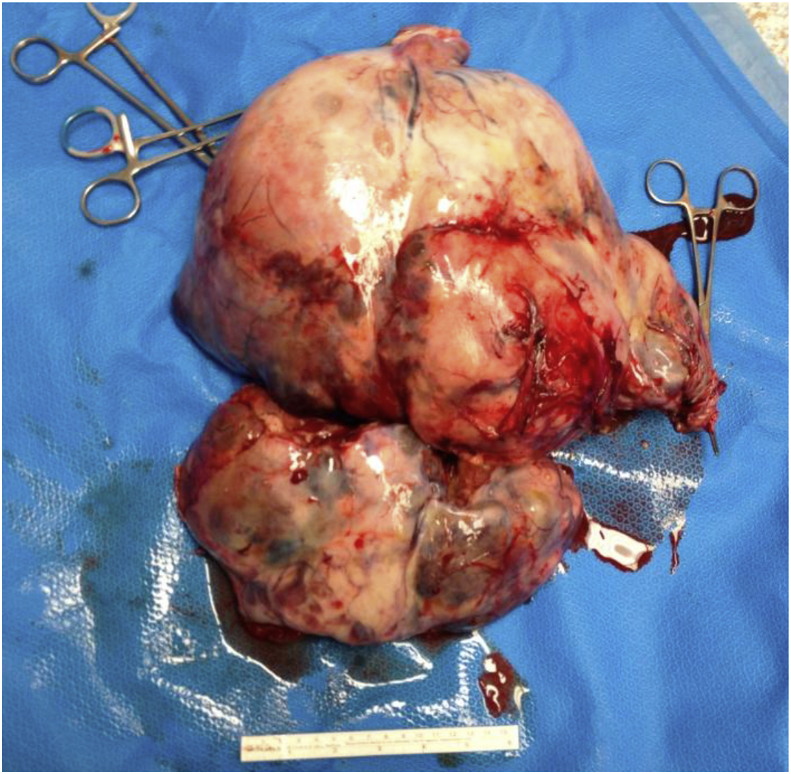
Operative specimen.

**Fig. 7:**
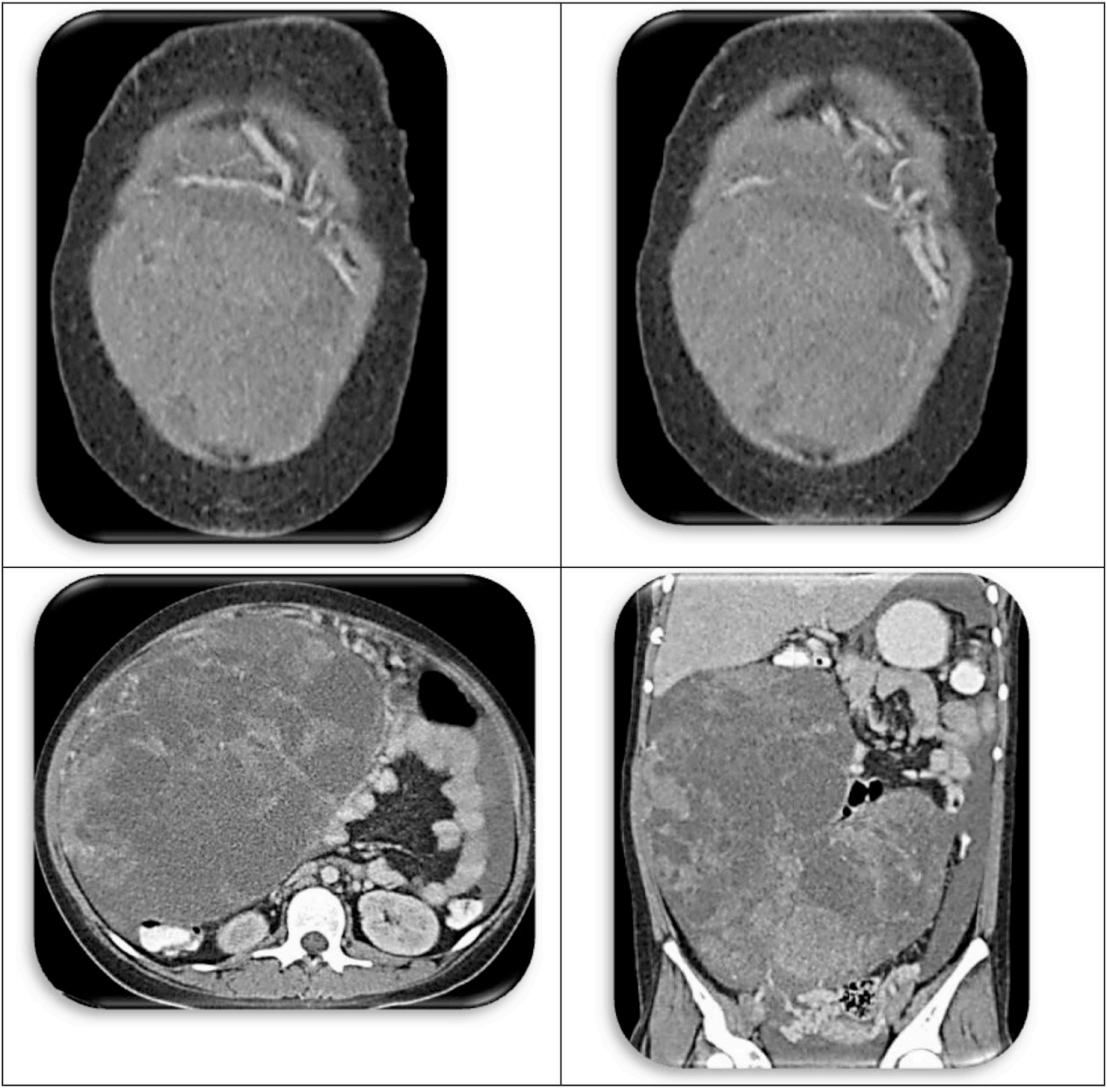
Scans of juvenile germ cell tumor.

A 13 years old pubertal young lady presented with abdominal distension and discomfort, and isosexual precocious puberty. The tumor was huge (33 × 15.5 × 33 cm), and it was adherent to the surrounding structures (Figs. 6, 7). As the dissection progressed inferiorly and laterally, the operative incision was stretched open to provide better exposure. Pulling the retroperitoneum tore the tumor capsule and caused torrential hemorrhage. Fortunately, most of the omental vessels had been ligated, and the bleeding was arrested expeditiously by controlling the ovarian pedicle.

This young lady was followed post-operatively with tumor markers and imaging. Her tumor never recurred, which is consistent with the observation that syndromic patients, even those with tumor rupture, have an excellent prognosis.

### 3.1 Discussion of second case

What triggers tumor neovascularity, the ingrowth of omental blood vessels into certain tumors, notably ovarian tumors and uterine fibroids [15]? Perhaps rapid growth of the tumor exceeds its blood supply; the resultant ischemia causes release of angiogenic mediators.

The omentum is termed “policeman of the abdomen”! In laparoscopy, we are taught to “follow the omentum” to the pathology! It is indeed a remarkable organ, derived from mesothelial cells, consisting of adiposites and lymphoid aggregates. Omental lymphatics filter antigens and pathogens from ascitic fluid, a process vital to developing immunity and protecting the peritoneal cavity. Chemotactic stimuli lead the omentum to foci of inflammation, where recruitment of inflammatory cells (lymphocytes and phagocytes) combat infection. Stem cells promote wound healing by angiogenesis and fibrosis. Metastatic cells are filtered so effectively that omental lymphatics may be clogged by tumor cells [16].

**Fig. 8:**
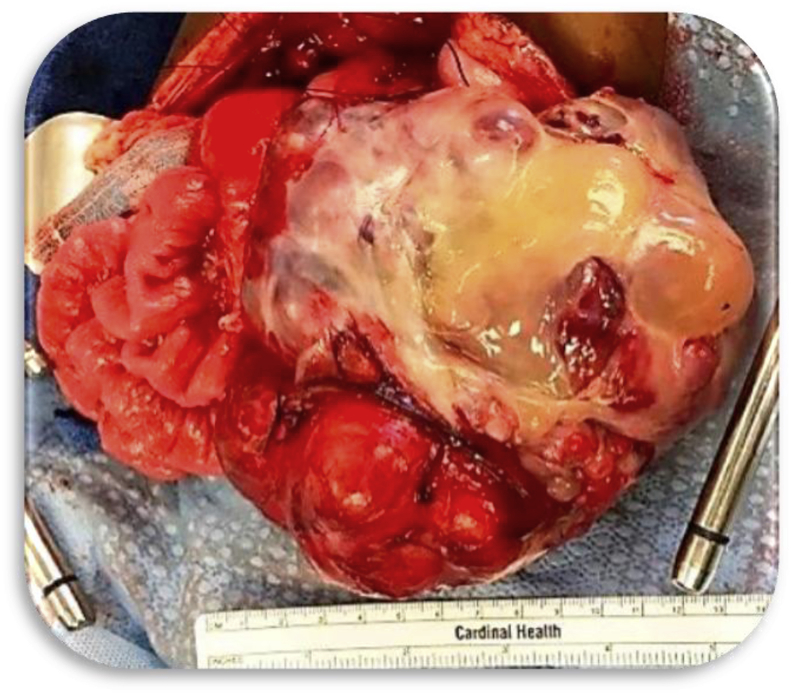
Operative specimen.

Case 3 A Teratoma that Eroded into the Stomach (Fig. 8)

**Fig. 9:**
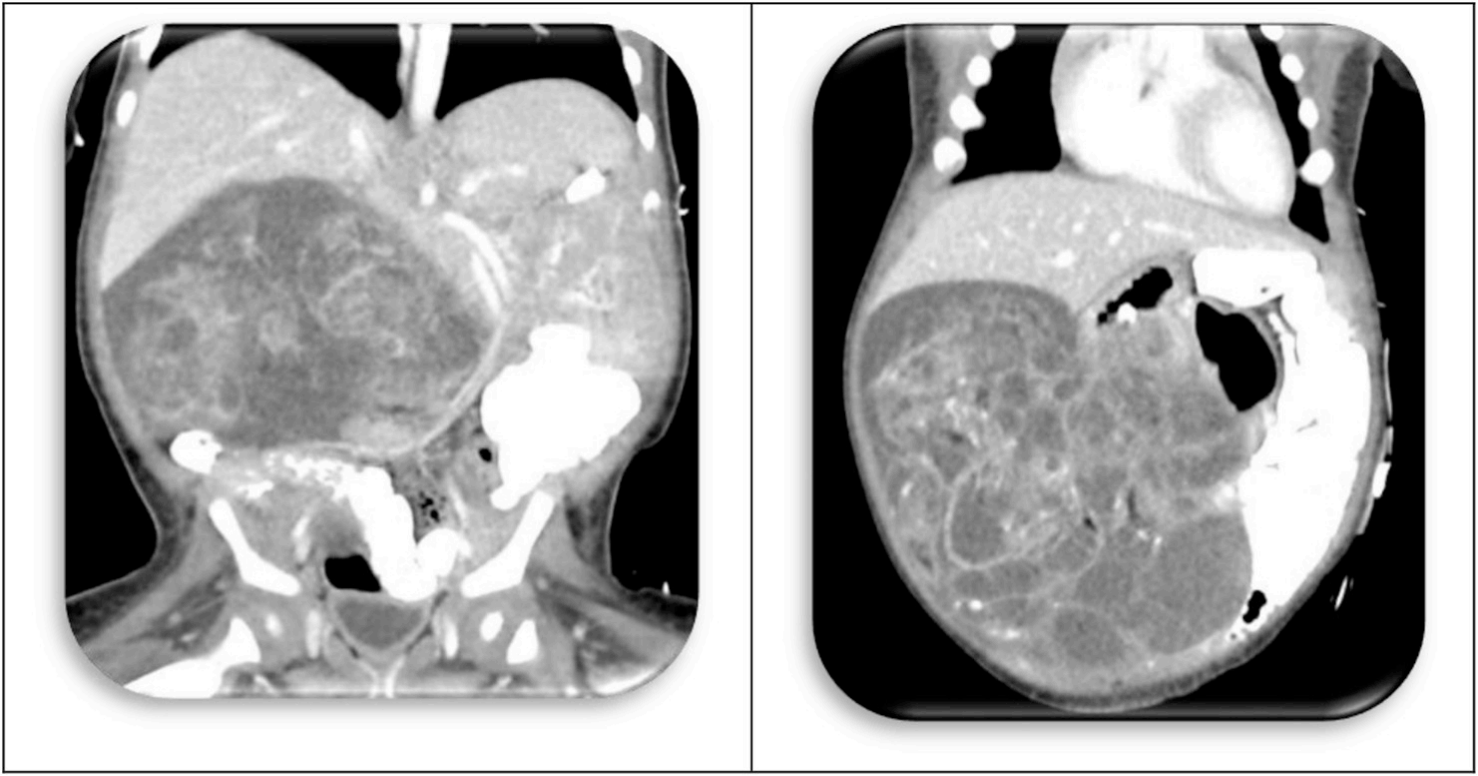
CT scan of Gastric Teratoma.

A 9 months old boy presented with abdominal distension, hematemesis, and profound anemia (HGB 2.7/HCT 12.2). He received 5 units of blood pre-operatively. He had a huge teratoma that was attached to the caudate lobe of the liver and the antrum of the stomach. The tumor had eroded through the stomach wall, causing hemorrhage and leakage of gastric contents into the tumor (Figs. 8, 9) [[17], [18], [19]].

**Fig. 10:**
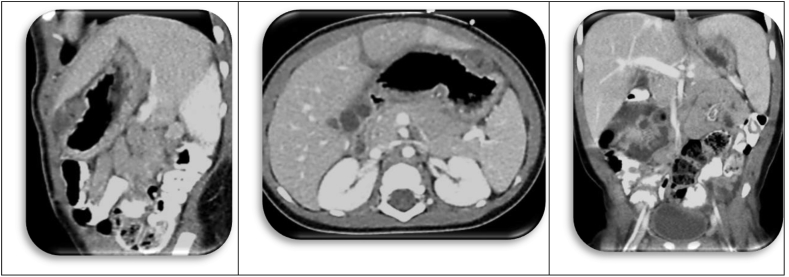
CT scan Showing Recurrence in Stomach and Liver.

Pathology was Mature Teratoma with islands of Yolk Sac Tumor. The child was not treated with chemotherapy in accordance with principle #3 above. The tumor did recur where it was originally attached (Fig. 10), but the histology was MT (not YST). These recurrences were excised and never recurred.

Case 4 Infant with Mesenteric Teratoma

**Fig. 11:**
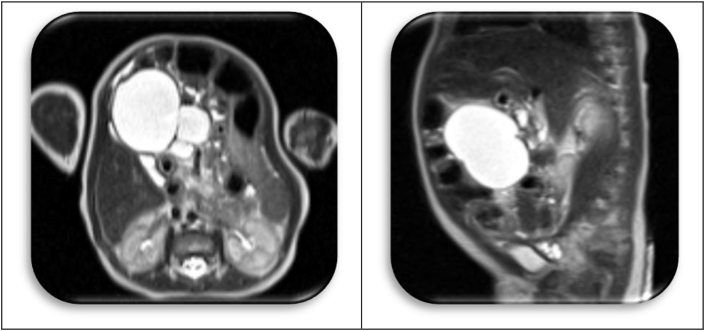
MR.

The radiographic (MR) diagnosis in this infant was “mesenteric cyst” (Fig. 11). The excision was uneventful, but the pathology finding was unexpected: Grade I Immature teratoma without malignant elements. There was never any recurrence.

## 4 Discussion of cases 3 and 4

Retroperitoneal teratomas are notorious for distorting or obliterating the vascular anatomy. The renal vessels may be splayed apart; and/or the vena cava and/or portal vein may be encased by tumor, making resection particularly difficult [20,21]. Taking a cue from hepatoblastoma surgery, it was hoped that pre-operative chemotherapy would shrink the tumor and facilitate removal. Unfortunately the desired effect was not achieved. Chemotherapy effectively destroyed the malignant elements, but allowed unfettered growth of the immature teratoma, termed a “growing teratoma” (Principle #5, above).

The location of our tumors was intra-abdominal rather than retroperitoneal. The anatomy was distorted by their large size, but the vasculature was displaced rather than obscured, and their resection was straight-forward.

Case 5 Metastatic Gestational Choriocarcinoma

**Fig. 12:**
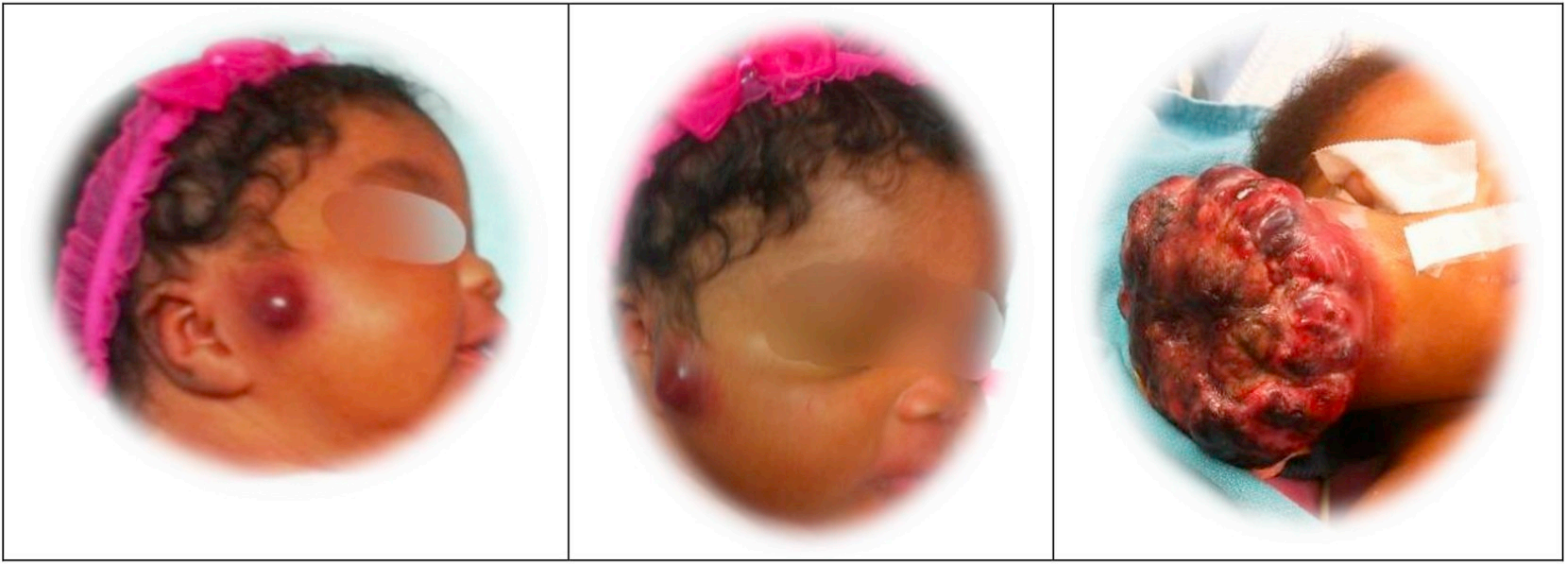
Photos of infant.

**Fig. 13:**
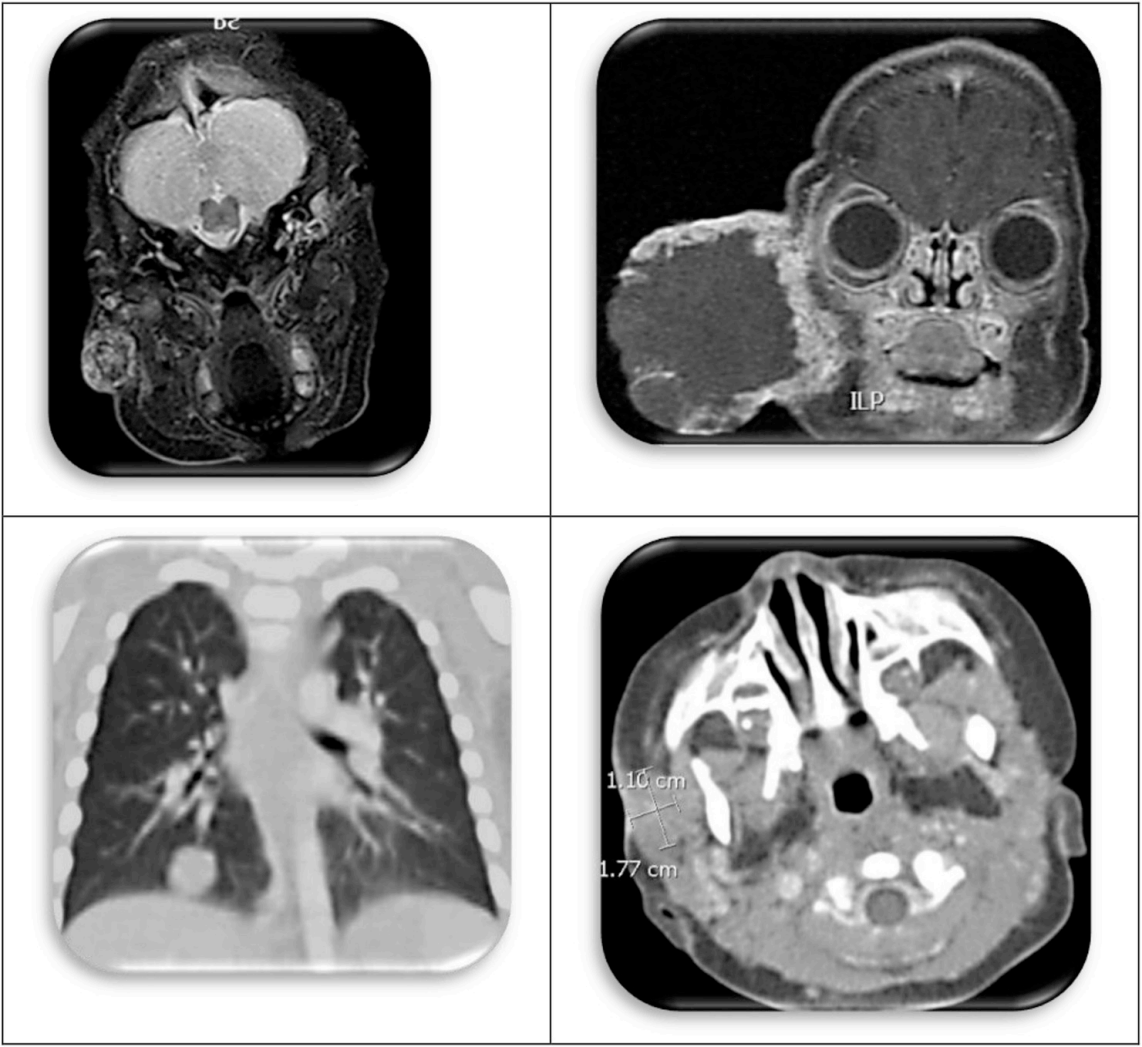
Mr of the choriocarcinoma.

This baby's mother brought her to the emergency department, because of the growth on her cheek.(Fig. 12). Surgery was consulted for drainage of an “abscess”. The photos were taken for consultation with an oncologist and otolaryngologist. An MR was obtained and the mass was thought to be a “vascular malformation”. She was admitted and treated with propranolol and prednisone, and the lump transiently diminished in size; however, the mass ulcerated and bled, leading to readmission and transfusion; ultimately, she was referred to another institution for embolization. Unfortunately, she was lost to follow-up for a time; and when she reappeared, the tumor had grown to monstrous proportions (Fig. 13). Obviously, the initial diagnosis of vascular anomaly was erroneous. Biopsy revealed choriocarcinoma, and an elevated HCG and pulmonary metastasis. Her mother's HCG, also, was elevated, presumably from uterine involvement. Both mother and child responded well to chemotherapy and are disease free.

## 5 Discussion

This is a case of gestational choriocarcinoma arising in the mother's placenta, metastatic to the infant. Newborns with this disease usually present with anemia, hepatomegaly, and precocious puberty. Metastatic disease may involve the liver, lungs, brain, and skin [22].

Gestational choriocarcinoma occurs in 1/50,000 pregnancies. Non-gestational choriocarcinoma is even rarer; it arises from malignant degeneration of extra-embryonic germ cells in the brain, mediastinum, or gonads.

## 6 Conclusion

We learn from interesting cases, and Case 1 is illustrative of two errors that clinicians are especially prone to make:

1. Allowing emotion (rather than reason) to dictate therapeutic decisions
2. Jumping to conclusions (Cognitive Bias)

The oncologic data *is* ambiguous: adults with IT routinely receive chemotherapy, but children do not. Should an adolescent (a pubertal young lady) be treated as an adult or a child? Yet there is no evidence that IT responds to chemotherapy in either case. That conviction initially guided therapy; it was the best “evidence based treatment”. But the young lady's clinical course confounded the expectations of her physicians, causing them to question their initial decision. Considering her multiple recurrences, should not chemotherapy be tried?

The quandary for clinicians is that sometimes reappraisal and change is necessary; in other instances, the correct posture is to *stay the course*!

Clinicians are like detectives. What makes Sherlock Holms the master sleuth? His perception is more acute, and his conclusions are more accurate. A less competent detective jumps to conclusions, which inevitably do not take into account all of the facts. Once a theory is embraced, clues that contradict it are overlooked.

Clinicians do the same thing, as is demonstrated in Case 1. By (almost) all measures, the teratoma was becoming more mature (pathology, AFP levels, and gross appearance); however, this evidence was seemingly contradicted by the radiographs, which showed “persistent” or “recurrent” Immature Teratoma.

Choosing one explanation (assigning a label) obfuscates other possibilities.^1^ Our sure convictions cause us to overlook crucial bits of information. The evidence of increasing benignity was ignored, and a therapeutic approach was tailored to rid the patient – once and for all - of tumor. Teratomas may be metachronous and bilateral [17]. Sherlock Holms would have considered this fact and chosen a more nuanced surgical approach.

Mark Twain, “*It's not what we don't know that gets us into trouble. It's what we know for sure that just ain't so.”* Our certain conclusions may be “dead wrong” and cause us “double trouble”. The error is compounded by delayed recognition.

Case 5 reinforces the lesson that interpretations of radiographs may be flawed. No one wants to biopsy a vascular malformation, but correct diagnosis precedes appropriate therapy.

Interesting cases are engaging and memorable, and they illustrate important lessons:

• Once the best *evidence based therapy* is determined, “Stay the course!”
• Don't jump to conclusions. Make sure your solution to diagnostic dilemmas take into account all the facts.
• Don't take short cuts. Correct diagnosis always precedes effective therapy.

## Ethical approval

I have obtained IRB approval from both institutions in which these children were cared: University of South Alabama, Mobile AL and Palmetto Health Children's Hospital, Columbia, SC.

## Sources of funding

Myself.

## Author contribution

Dr. Nottingham was the sponsoring author in Columbia. He enabled me to access patient information.

James M. Nottingham, MD, FACS.

Professor of Surgery, University of South Carolina School of Medicine.

2 Richland Medical Park, Suite 300.

Columbia, South Carolina 29203.

Michael E. Haney, M.E. helped obtain IRB approval in SC.

The pathology photomicrographs were provided by Elizabeth A. Manci, MD, who is an attending pathologist at Children's and Women's Hospital in Mobile.

## Conflicts of interest

None.

## Research registry number

4367.

## Trial registry number

None.

## Guarantor

I do. James G. Glasser, MD.

## Appendix A Supplementary data

Process Checklist Process Checklist

Addendum_V2 Addendum_V2

## Footnotes

1 Einstein famously introduced a “fudge factor” into his gravitational equations to prevent the universe from imploding! The facts were made to conform to the theory rather than vice versa.
