# Supplementary material for: Case series: Five pediatric germ cell/sex cord stroma tumors
Source: Ann Med Surg (Lond). 2018 Nov 28;37:11–20. doi: 10.1016/j.amsu.2018.11.011 (PMC6287085; doi:10.1016/j.amsu.2018.11.011)
Supplement: Process Checklist [file mmc1.docx]

| PROCESS CHECKLIST | | | |
| --- | --- | --- | --- |
| Section | **Item** | **Checklist Description** | **Page Number** |
| Title | 1 | The words “case series” and the area of focus should appear in the title (e.g. disease, exposure/intervention or outcome).  Case Series: Five Paediatric Germ Cell/Sex Cord Stroma Tumours | 1 |
| Abstract | 2a | **Introduction:** What is the unifying theme of the case series?  It is a group of unusual paediatric ovarian tumours. | Introduction  Done  Done  Done |
|  | 2b | **Methods**: Describe what was done, how and when was it done and by whom.  The principal author operated upon these children (at two institutions). Post-operative management was in conjunction with the oncologists, who because of the unusual nature of these cases sought the assistance of acknowledged experts (tertiary referral centres). |  |
|  | 2c | **Results:** What was found?  Management was frequently counter-intuitive; however, all of these children are doing well. |  |
|  | 2d | **Conclusion:** What have we learned and what does it mean.  These are idiosyncratic tumours; “one size does not fit all”. Perhaps in managing these tumours more vigilance and tailored (individualized) therapy is necessary. |  |
| Introduction | 3 | Explain the scientific background and rationale for the case series. What is the unifying theme - common disease, exposure, intervention and outcome? Why is this study needed?  Because of the unusual nature of these tumours; and especially in case #1, a cognitive bias over-ruled careful attention to all facts – despite having expert guidance in management. | Done |
| Methods | 4a | **Registration and ethics:** State the research registry number in accordance with the declaration of Helsinki - "Every research study involving human subjects must be registered in a publicly accessible database before recruitment of the first subject" (this can be obtained from; ResearchRegistry.com or ClinicalTrials.gov or ISRCTN). Even retrospective studies should be registered prior to submission. State whether ethical approval was needed and if so, what the relevant judgement reference from the IRB or local ethics committee was? If ethical approval was not needed, state why. | IRB approval was obtained from both institutions: Palmetto Health and University of South Alabama  Research Registry: #4367. |
|  | 4b | **Study design** - State the study is a case series and whether prospective or retrospective in design, whether single or multi-centre and whether cases are consecutive or non-consecutive.  It is a retrospective Case Series that is non-consecutive. | Done |
|  | 4c | **Setting**: Describe the setting(s) and nature of the institution in which the patient was managed; academic, community or private practice setting? Location(s), and relevant dates, including periods of recruitment, exposure, follow-up, and data collection.  Two academic institutions: Palmetto Health Children’s Hospital in Columbia, SC and Children’s and Women’s Hospital, Mobile, AL. | Done |
|  | 4d | **Participants**: Describe the relevant characteristics of the participants (comorbidities, tumour staging, smoking status, etc.) State any eligibility (inclusion/exclusion) criteria and the sources and methods of selection of participants. Describe length and methods of follow-up.  The study consists of children/babies with unusual tumours of ovarian origin. The first case is the principal one. The other four cases are included to add interest and breadth to the discussion. | Done |
|  | 4e | **Pre-intervention considerations**: Patient optimisation: measures taken prior to surgery or other intervention e.g. treating hypothermia/hypovolaemia/hypotension in burns patients, ICU care for sepsis, dealing with anticoagulation/other medications and so on.  Each patient’s overall condition was optimized prior to surgery. Case #3 required transfusion of several units of blood pre-operatively. | Done |
|  | 4f | **Types of intervention(s) deployed**: To include reasoning behind treatment offered (pharmacological, surgical, physiotherapy, psychological, preventive) and concurrent treatments (antibiotics, analgesia, anti-emetics, nil by mouth, VTE prophylaxis, etc). Medical devices should have manufacturer and model specifically mentioned.  Surgery +/- Chemotherapy | Done |
|  | 4g | **Peri-intervention considerations**: Administration of intervention (what, where, when and how was it done, including details for surgery; anaesthesia, patient position, use of tourniquet and other relevant equipment, preparation used, sutures, devices, surgical stage (1 or 2 stage, etc.) and operative time. Pharmacological therapies should include formulation, dosage, strength, route and duration). Authors are encouraged to use figures, diagrams, photos, video and other multimedia to explain their intervention.  The indications for the interventions are documented by didactic tables and radiologic images. | Done |
|  | 4h | **Who performed the procedure(s)**: Operator experience (position on the learning curve for the technique if established, specialisation and prior relevant training).  The principal author (an senior paediatric surgeon) performed most (not all) of the surgery. | Done |
|  | 4i | **Quality control**: What measures were taken to reduce inter or intra-operator variation. What measures were taken to ensure quality and consistency in the delivery of the intervention e.g. independent observers, lymph node counts, etc  Guidance was sought in the management of these patients from acknowledged experts in the treatment of paediatric ovarian tumours. | Done |
|  | 4j | **Post-intervention considerations**: e.g. post-operative instructions and place of care. Important follow-up measures - diagnostic and other test results. Future surveillance requirements - e.g. imaging surveillance of endovascular aneurysm repair (EVAR) or clinical exam/ultrasound of regional lymph nodes for skin cancer.  Surveillance was by CT scan and MR and assessing tumour markers by blood tests | Done |
| Results | 5a | **Participants**: Report numbers involved and their characteristics (co-morbidities, tumour staging, smoking status, etc.).  There are 5 patients in this series: each with a different tumour each treated differently. | Done |
|  | 5b | **Changes;** Any changes in the interventions during the course of the case series (how has it evolved, been altered or tinkered with, what learning occurred, etc.) together with rationale and a diagram if appropriate. Degree of novelty for a surgical technique/device should be mentioned and a comment on learning curves should be made for new techniques/devices.  The manuscript discusses the changes that occurred during the course of treating patient #1’s multiple tumour recurrence (persistence).  Errors were made - “second guessing”, “cognitive bias” – and are mentioned not to criticise, but to highlight how even the best clinicians fall prey to these very human tendencies. | Done |
|  | 5c | **Outcomes and follow-up**  Clinician assessed and patient-reported outcomes (when appropriate) should be stated with inclusion of the time periods at which assessed. Relevant photographs/radiological images should be provided e.g. 12-month follow-up.  The outcome in all 5 patients was excellent. My view is that a correct evaluation of the situation prior to operation #4, might have allowed an “ovarian sparing” resection, rather than “radical extirpation”, in case #1.  As always, correct diagnosis informs correct therapy. | Done |
|  | 5d | **Intervention adherence/compliance and tolerability:** How was this assessed? Describe loss to follow-up (express as a percentage and a fraction) and any explanations for it.  Long term follow-up in all patients was obtained. |  |
|  | 5e | **Complications and adverse or unanticipated events**: Described in detail and ideally categorised in accordance with the Clavien-Dindo Classification. How they were prevented, mitigated, diagnosed and managed. Blood loss, wound complications, re-exploration/revision surgery, 30-day post-op and long-term morbidity/mortality may need to be specified.  This a major focus of the manuscript: human error, over-reliance upon our very accurate imaging technology (MRI). | Done |
| Discussion | 6a | **Summarise key results:**  We all learn from “interesting cases”. Hopefully, clinicians’ insight will be sharpened by the presentation of these cases.   1. We are prone to label, to jump to conclusions, and ignore contrary facts. 2. We believe our impressions and resist unpleasant measures to verify them. 3. We worry, “develop cold feet”, when things go different than expected. This is the opposite error to #1.   When a diagnosis is legitimately established and the best therapy selected, “stay the course”; don’t abandon ship! | Done |
|  | 6b | **Discussion of relevance:** Relevant literature, implications for clinical practice guidelines, how have the indications for a new technique/device been refined and how do outcomes compare with established therapies and the prevailing gold standard should one exist and any relevant hypothesis generation.  See above (6a) | Done |
|  | 6c | **Strengths and limitations of the study**  Although the appropriate treatment of Immature Teratoma in adolescence is still unresolved; and hopefully, this case presentation will further resolution of this quandary, in that the manuscript identifies “blind spots” in clinical practice, I believe it has relevance beyond this particular problem. | Done |
|  | 6d | **The rationale for any conclusions?**  as above, 6c | Done |
| Conclusions | 7a | **State the key conclusions from the study**  as above, 6a, 6c | Done |
|  | 7b | **State what needs to be done next, further research with what study design.**  The “experts” who assisted in the management of case #1 are accruing a series of adolescents with pure Immature Teratoma to determine if other patients follow the same clinical course as our patient. Does the “growing teratoma” syndrome ever occur in these older patients (adolescents or adults)? If so, that would mitigate against chemotherapy being effective in any patient with an Immature Teratoma.  Is there evidence that chemotherapy actually hastens maturation of Immature Teratomas? |  |
| Additional Information | 8a | **State any conflicts of interest** | None |
|  | 8b | **State any sources of funding** | None besides myself |
