# Supplementary material for: Case series: Five pediatric germ cell/sex cord stroma tumors
Source: Ann Med Surg (Lond). 2018 Nov 28;37:11–20. doi: 10.1016/j.amsu.2018.11.011 (PMC6287085; doi:10.1016/j.amsu.2018.11.011)
Supplement: Addendum_V2 [file mmc2.docx]

**Addendum**

**Table 2:** Definition of Terms

| Stage |  | Grade |  |
| --- | --- | --- | --- |
| 1 | Tumor in one ovary, completely resected | 1 | IT in < 1 low power (microscopic) field |
| 2 | Incomplete resection; residual tumor | 2 | IT in 1-3 low power (microscopic) fields |
| 3 | Spread confined to the peritoneal cavity (LN’s, omentum, ascites) | 3 | IT in >3 low power (microscopic) fields |
| 4 | Distant spread (liver) |  |  |

Summary of **Immature Teratoma** Treatment Data:

Children (n= 44) completely resected **IT**

- 31/44 pure IT
- 13/44 had microscopic foci of **YST**
- Grade I (17/44)
- Grade II (12/44)
- Grade III (2/44)
- *Gliomatosis Peritonei*, Stage III (12/44)
- Nodal Gliomatosis in 3/16 patients sampled

EFS = 98%, OS = 100% - regardless of Grade or presence of microscopic foci of **YST**

Patients (n=179) with pure **IT**

- 98/179 Pediatric (mean age 10)
- 81/179 Adult, Rx surgery + post-operative chemotherapy
- 90/98 pediatric patients Rx surgery
- 60 patients were Stage I (Rx Surgery)
- 30 patients were Stage II (Rx Surgery)
- 8 patients were Stage III (Rx Surgery + post-operative chemotherapy)
- AFP  => 83-183 ng/mL
- **9/98** Pediatric patients (9%) relapsed, with follow-up of approximately 7 years
- **8/38** Grade III patients (21%) relapsed
- Post-op chemotherapy (Cisplatin, Etoposide, Bleomycin)did not prevent relapse
- Grade III, Stage I/II = 92% EFS; Grade III, Stage III EFS = 52%
- OS of all Grade III children (regardless of Stage) was 100%
- Salvage, in some patients, was achieved by surgery alone
- Risk Factor Analysis -> **Grade of tumor and completeness of excision are the most important factors in predicting tumor relapse [17].**

**Table 10:** Treatment of Immature Teratoma [2]

|  |  | Relapse  Overall | EFS  Overall | EFS  Stage I | EFS  Stage II | EFS Stage III | EFS  Stage IV | OS |
| --- | --- | --- | --- | --- | --- | --- | --- | --- |
| **#** Patients  **Pediatric**  Adult | **98** children  81 adult | **9%**  14% | **91%**  87% |  |  |  |  | **100%**  93% |
| **Age** (mean)  **Pediatric**  Adult | **10 years**  26 years |  |  |  |  |  |  |  |
| **%** Patients  **Pediatric**  Adult |  |  |  | **60%**  53% | 12%  6% | **28%**  33% | **0**  7% |  |
| Grade I  **Pediatric**  Adult | **31%**  9% | **0%**  0% |  |  |  |  |  | **100%**  91% |
| Grade II  **Pediatric**  Adult | **20%**  33% | **0%**  4% |  |  |  |  |  | **100%**  91% |
| Grade III  **Pediatric**  Adult | **39%**  56% | **21%**  20% |  | **92%**  91% | 92%  91% | **52%**  65% | 65% | **100%**  88% |
| Lost to Follow-up  **Pediatric**  Adult | **10%**  2% |  |  |  |  |  |  |  |
| **AFP**  Adults only | 46% normal  45% elevated |  |  |  |  |  |  |  |
| Surgery  only  **Pediatric** | **92%** |  |  |  |  |  |  |  |
| Surgery + Chemotherapy  **Pediatric**  Adult | **8%**  100% |  |  |  |  |  |  |  |
